# Hypertonic glucose vs insulin–dextrose to prevent hypoglycaemia following treatment for hyperkalaemia (HIGH-K): protocol for a double-blind randomized controlled trial

**DOI:** 10.1080/07853890.2026.2701477

**Published:** 2026-07-26

**Authors:** Samuel Ford, Adam La Caze, Ian Coombes, Angela Hills, Jaimi Greenslade, Julian Williams

**Affiliations:** ^a^ Faculty of Health, Medicine and Behavioural Sciences, The University of Queensland, Australia; ^b^ Pharmacy Department, Royal Brisbane and Women’s Hospital, Brisbane, Australia; ^c^ Emergency and Trauma Centre, Royal Brisbane and Women’s Hospital, Brisbane, Australia; ^d^ Faculty of Health, School of Clinical Sciences, Queensland University of Technology, Australia

**Keywords:** Emergency department, endogenous insulin, hyperkalaemia, hypoglycaemia, hypertonic glucose solutions, randomised controlled Trials

## Abstract

**Background:**

Hyperkalaemia is a life-threatening electrolyte abnormality commonly managed with intravenous insulin–dextrose therapy (IDT). Although effective, IDT frequently causes hypoglycaemia, particularly in patients without diabetes. Glucose-only therapy, which leverages endogenous insulin production, may offer comparable potassium-lowering effects with reduced hypoglycaemia risk. However, evidence remains limited.

**Methods:**

The HIGH-K Trial is a single-centre, double-blind, randomised controlled trial in adult, non-diabetic patients presenting to an Australian Emergency Department with hyperkalaemia (>5.5 mmol/L [99 mg/dL]). Ninety-five participants are randomised 1:1 to receive either glucose-only therapy (100 mL 50% dextrose bolus followed by 250 mL 10% dextrose infusion over 2 h) or standard IDT (10 units IV insulin with 25 g dextrose followed by 250 mL saline infusion). The primary safety outcome is the incidence of hypoglycaemia (<3.9 mmol/L [70 mg/dL]) within six hours. The primary non-inferiority outcome is the mean change in serum potassium from baseline to two hours, using a non-inferiority margin of −0.5 mmol/L (–9 mg/dL). Secondary outcomes include severity of hypoglycaemia, rescue insulin requirements, and serum insulin/C-peptide levels.

**Discussion:**

This is the first double-blind, randomised controlled trial to directly compare the safety and biochemical non-inferiority of glucose-only therapy versus standard insulin–dextrose therapy in the emergency department. By utilising a continuous glucose infusion following a bolus, the protocol aims to sustain endogenous insulin release and optimise intracellular potassium shift while preventing hypoglycaemia. If non-inferiority is demonstrated, this approach could provide a safer alternative in high-acuity or resource-limited clinical settings.

**Conclusion:**

Results will be disseminated in peer-reviewed journals and at national and international conferences. Findings may inform future research and clinical practice guidelines regarding glucose-only therapy for hyperkalaemia.

## Administrative information

The trial has been prospectively registered with the Australian New Zealand Clinical Trials Registry (ACTRN12625000617460). This work was supported by the Emergency Medicine Foundation (EMF) through a Leading-Edge Grant (EMLE-306R43-2025-FORD). EMF provided expert peer review of the protocol and methodology as part of the competitive grant application process but has no ongoing role in the conduct, analysis, or reporting of the trial.

## Introduction

Hyperkalaemia is a potentially life-threatening electrolyte disorder associated with cardiac arrhythmias and cardiac arrest [1]. Insulin–dextrose therapy (IDT) remains the mainstay of acute management, promoting intracellular potassium uptake *via* stimulation of the Na^+^–K^+^–ATPase pump. Despite co-administration of dextrose, IDT carries a significant risk of iatrogenic hypoglycaemia, with an estimated incidence of 17.2% [2]. Locally, rates as high as 30% have been observed, including 6.7% classified as severe hypoglycaemia [3].

The regimen of 10 units of short-acting insulin co-administered with 25 g of glucose has remained the standard of care for decades, despite being derived primarily from small studies in haemodialysis populations [4]. There is broad agreement that this regimen warrants re-evaluation to minimise the risk of iatrogenic hypoglycaemia. Some studies have found that lower dose insulin (<10 units) reduces hypoglycaemia risk without compromising potassium-lowering efficacy, while others have demonstrated a diminished biochemical response and no difference in hypoglycaemia [2,5]. Dextrose dosing is similarly conflicting, but doses exceeding 25 g may be associated with a lower risk of hypoglycaemia [5]. The delivery method also appears important with some research suggesting a reduced risk of hypoglycaemia when dextrose is provided as a sustained infusion rather than bolus administration [2]. Most of this evidence is derived from retrospective studies, and to date, no randomised controlled trials (RCT) have directly examined the optimal insulin-to-glucose ratio for the management of hyperkalaemia.

In the absence of a definitive ratio, clinicians must instead rely on patient-specific risk factors to guide therapy. Lower pre-treatment blood glucose level (BGL) is the strongest predictor of hypoglycaemia following IDT, with non-diabetic patients particularly susceptible [2,6,7]. Other potential risk factors include renal insufficiency, older age and lower body weight [6].

The UK Kidney Association (UKKA) has recently reviewed this body of evidence and recommends 10 units of insulin with 25 g of glucose, followed by a 5-hour infusion of 250 mL of 10% glucose when the pre-treatment blood glucose concentration is less than 7 mmol/L (126 mg/dL) [8]. Randomized controlled trials are required to determine whether tailoring therapy based solely on pre-treatment glucose concentration is sufficient, or whether additional risk factors should inform individualised treatment strategies.

As an alternative to insulin-based therapy, glucose-only therapy has been proposed as an approach in patients without diabetes [9,10]. The proposed mechanism is that endogenous insulin release following glucose administration upregulates the Na^+^–K^+^–ATPase pump and drives potassium intracellularly. While theoretically sound, the UKKA currently advises against this strategy due to concerns that hypertonic glucose may exacerbate hyperkalaemia through osmotic solute drag and that endogenous insulin release may be insufficient to achieve a clinically meaningful reduction in serum potassium concentration [8]. Our recently published scoping review sought to systematically evaluate the effect of glucose administration on potassium concentrations [11]. The review found that while there is evidence for increased potassium in response to hypertonic glucose, this predominantly occurs in patients with diabetes, particularly type 1 diabetes. It appears that in patients with diabetes, pathophysiological changes of insulin insufficiency, resistance, and hypoaldosteronism result in an inability to prevent hyperosmolar induced hyperkalaemia in some patients [12–16].

Most studies in patients without diabetes found reductions in potassium following glucose administration, ranging from 0.15 mmol/L (2.7 mg/dL) to 0.91 mmol/L (16.4 mg/dL). The most robust evidence comes from a small randomised controlled trial by Chothia et al. which found average potassium reductions of 0.5 mmol/L (9 mg/dL) in the glucose-only arm compared to 0.83 mmol/L (15 mg/dL) in the IDT arm [9]. Importantly, the lower potassium reduction was offset by an improved safety profile as no hypoglycaemia occurred in the glucose-only group, compared to 20% in the IDT group. Additional support is provided by the study by DeFronzo et al. which demonstrated a 0.81 mmol/L (14.6 mg/dL) reduction in plasma potassium following a two-hour glucose infusion where blood glucose was maintained at 6.9 mmol/L (124 mg/dL) above baseline in healthy participants [17].

Endogenous insulin production following glucose administration is dose dependent and the hypokalaemic effect is dependent on the insulin concentrations achieved [18–20]. There does, however, appear to be a ceiling to the hypokalaemic effect [17]. Studies measuring insulin levels after glucose administration consistently report lower insulin levels compared to studies examining IDT. Most studies, however, used bolus glucose administration, and we hypothesise that a glucose bolus followed by a continuous infusion will sustain higher insulin levels over time and enhance potassium-lowering efficacy [11]. Since endogenous insulin release may be suppressed by counter-regulatory hormones during critical illness, our study protocol restricts enrollment to hemodynamically stable patients [21].

This trial responds to calls for glucose-only therapy to be included in future clinical trials for non-diabetic patients with hyperkalaemia [10]. The cross-over trial by Chothia et al. provides an important proof of concept but is limited by its small sample size and specific patient population, being restricted to haemodialysis recipients [9]. Our study aims to build on this foundation by enrolling a larger and more diverse ED population and adding nebulized salbutamol to both treatment arms to exploit complementary mechanisms for intracellular potassium shift. Furthermore, in the glucose-only arm, participants receive a two-hour glucose infusion following the initial bolus. This may sustain endogenous insulin release, thereby increasing the total potassium shift.

## Aims and objectives

The aim of the HIGH K Trial is to determine the safety and noninferiority of glucose-only therapy compared with insulin–dextrose therapy for the treatment of hyperkalaemia in adult, non-diabetic ED patients. The primary safety objective is to compare the incidence of hypoglycaemia (BGL <3.9 mmol/L [70 mg/dL]) within six hours post-treatment between groups. The primary non-inferiority objective is to compare mean change in serum potassium from baseline to two hours between groups.

Secondary objectives are to assess hypoglycaemia severity, determine proportion requiring rescue insulin therapy, evaluate ECG resolution and compare serum insulin and C-peptide levels at key time points.

## Patients and methods

### Design and setting

This trial is a single-centre, double-blind, parallel-group, randomised controlled trial. It involves adult patients presenting to the ED with hyperkalaemia and will be conducted from November 2025 to November 2027.

### Screening, eligibility and consent

**Figure 1.**
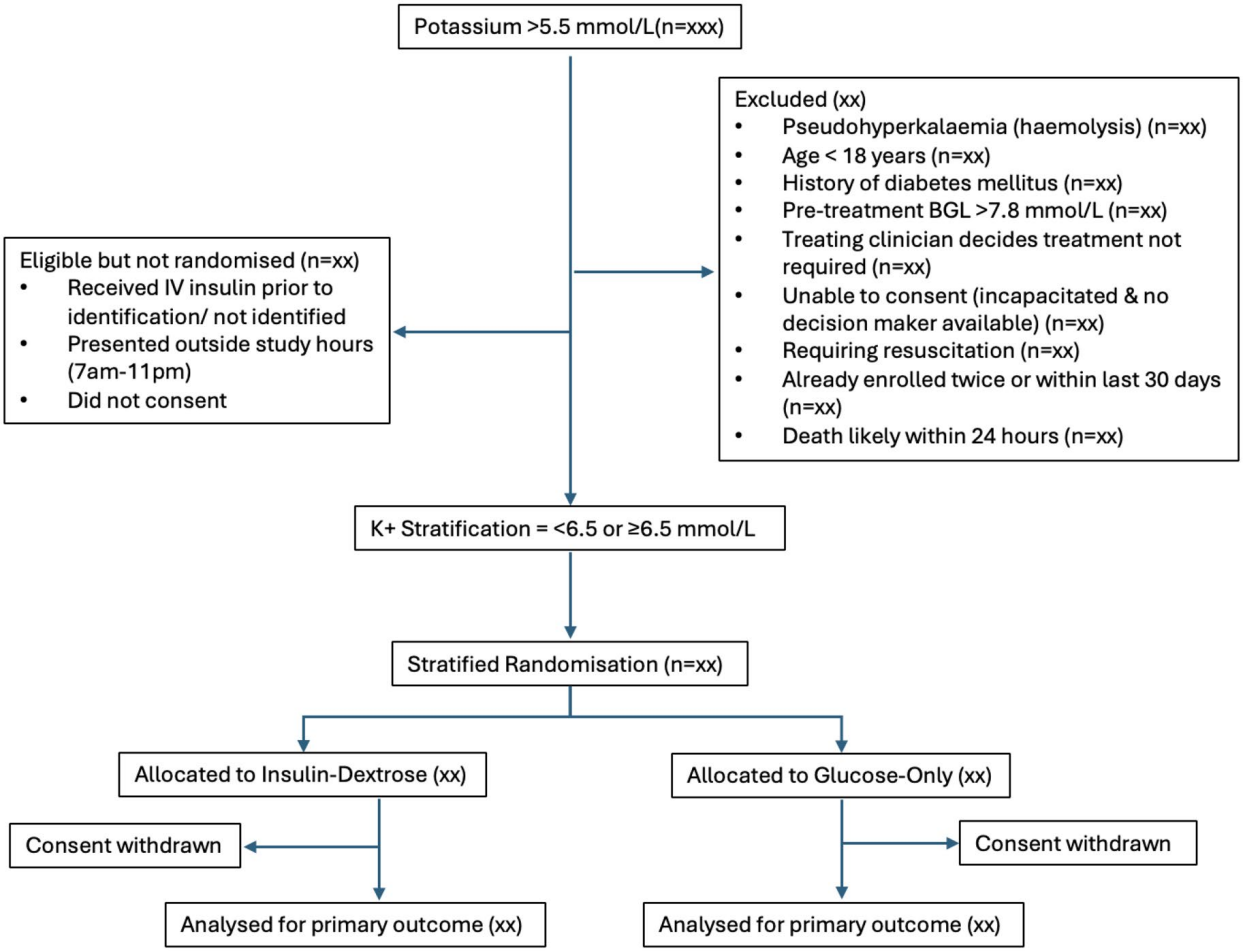
CONSORT diagram of participants in the HIGH K trial. HIGH K, Hypertonic glucose vs Insulin-dextrose to prevent HypoGlycaemia following treatment for hyperKalaemia; CONSORT Consolidated Standards of Reporting Trials; BGL, blood glucose level; IV, intravenous.

Potential participants presenting with hyperkalaemia are identified and screened for eligibility between 07:00 and 23:00, coinciding with clinical pharmacist availability for blinded infusion preparation. Inclusion and exclusion criteria are outlined in Table 1. The flow of participants through the screening and recruitment process is detailed in Figure 1. Informed consent will be obtained by members of the research team or ED clinical pharmacists trained in trial procedures. Eligible participants are provided with study information and given up to 15 min to consider participation.

**Table 1.** Study inclusion and exclusion criteria.

Inclusion criteria	Exclusion criteria
1.Age >18 years.	1.History of diabetes mellitus or pre-treatment BGL >7.8 mmol/L (140 mg/dL).
2.Blood gas **or** serum potassium >5.5 mmol/L (99 mg/dL).	2.Requiring resuscitation (cardiac arrest, unstable arrhythmias, SBP <90mmHg, HR <45 BPM).
3.Treating clinician determines need for active treatment and agrees to enrol patient in the trial.	3.Intravenous insulin administered prior to randomisation.
4.Ability to provide informed consent or consent obtained by authorised substitute decision-maker.	4.Enrolled in the trial within the past 30 days or have previously been enrolled more than once.
	5.Suspected or confirmed intracranial/ intraspinal ischaemic or haemorrhagic stroke.
	6.Known glucose-galactose malabsorption syndrome.
	7.Known allergy to corn (maize) and corn products.

BGL: blood glucose level; SBP: systolic blood pressure; mmHg: millimetres of mercury; BPM: beats per minute.

Standard consent is provided in written form. However, if a patient is unable to provide a signature due to acute illness or the requirement for contact precautions (e.g. respiratory isolation), verbal consent will be obtained. In such instances, the verbal agreement must be witnessed and signed by two independent staff members to ensure procedural integrity. If a patient lacks decision-making capacity, written consent may be obtained from an authorised substitute decision-maker. Patients enrolled *via* a substitute decision-maker will be approached once capacity returns and offered the opportunity to either provide ongoing informed consent to continue in the study or withdraw their participation.

### Protocol amendments

Two formal amendments to the HREC-approved protocol have been implemented since trial commencement. Amendment 1 (approved 5/11/25) updated the patient consent procedure to permit verbal consent process discussed above.

Amendment 2 (approved 11/5/26) added serum C-peptide as a co-collected secondary/mechanistic endpoint at baseline, 1 h, and 2 h post-treatment, to provide a direct measure of endogenous insulin secretion and enable mechanistic characterisation of the glucose-only intervention. Participants enrolled prior to Amendment 2 will not have C-peptide samples collected; C-peptide analyses will therefore be restricted to eligible participants, and this will be acknowledged as a limitation in the final publication.

Protocol modifications are communicated to relevant parties *via* email and face-to-face follow up. The trial registration record (ACTRN12625000617460) has been updated accordingly.

### Intervention

Participants will be allocated *via* stratified (potassium <6.5 mmol/L [117 mg/dL] or ≥6.5 mmol/L [117 mg/dL), block randomisation to receive either glucose-only therapy or insulin–dextrose therapy (IDT) in a 1:1 ratio with the use of sequential, opaque, sealed envelopes. Envelopes are opened by members of the research team or clinical pharmacists only after formal participant enrolment is confirmed and documented. Interventions will be prepared by the same staff who open the envelope and away from clinical areas. Study solutions will appear visually identical to ensure blinding of participants and treating clinicians. The study period extends for six hours following randomisation. In eligible participants, treatment commences as soon as possible after enrolment.

In the glucose-only arm, participants will receive 100 mL of 50% dextrose administered over 10 min, followed immediately by 250 mL of 10% dextrose infused over two hours.

In the insulin–dextrose arm, participants will receive 10 units of intravenous insulin (Actrapid) mixed with 50 mL of 50% dextrose and 50 mL of 0.9% sodium chloride (total 100 mL), administered over 10 min, followed by 250 mL of 0.9% sodium chloride infused over two hours.

Infusion bag 1 (100 mL) will be prepared by modifying 0.9% sodium chloride bags according to the assigned intervention. Study labels will be affixed to the front of each bag. Infusion bag 2 (250 mL) will consist of the proprietary product used in each arm without alteration; these bags will be concealed using an opaque black sleeve to mask product identity, with study labels applied to the exterior.

Calcium gluconate (10 mL = 2.2 mmol [40 mg/dL]) and nebulised salbutamol (10 mg) will be administered to all participants, either before or after randomisation. While calcium is generally only recommended for patients with severe hyperkalaemia or those with ECG changes, a decision to include it for all randomised participants was made to prioritize participant safety in the ED setting. Up to 50% of patients with hyperkalaemia do not exhibit ECG changes [1,22]. Additionally, ECG interpretation often requires access to baseline ECG recordings which may not be available or accessible in the ED setting. The potential benefits of early cardiac stabilisation with calcium gluconate outweigh the risks associated with administration in patients without ECG changes, particularly in the context of a clinical trial. Adverse events related to its use have been shown to be very rare [8]. Additional therapies such as repeat calcium doses, potassium binders, or sodium bicarbonate may be used at the treating clinician’s discretion. All medications are administered *via* peripheral intravenous access. Clinicians should exercise appropriate care, as both hypertonic glucose and calcium gluconate carry a risk of thrombophlebitis and extravasation when administered peripherally. These adverse events are prospectively captured as secondary safety outcomes.

Throughout the six-hour intervention period, clinical reassessment will occur at regular intervals in accordance with routine ED practice and the study monitoring schedule. If potassium levels are unchanged or have increased at two hours post-study intervention, then the treating clinician may administer “rescue” insulin. Rescue insulin will also be given at any time in the event of clinical deterioration (e.g. worsening ECG changes or haemodynamic instability). Administration of rescue insulin does not preclude or delay escalation to renal replacement therapy, which remains at the treating team’s discretion and follows standard institutional protocols. Both rescue insulin administration and dialysis commencement are recorded as study events. Senior ED medical staff may unblind participants to manage clinical deterioration. Additionally, inpatient medical teams may request unblinding after the six-hour study period if the information is necessary for ongoing care.

### Follow-up

**Figure 2.**
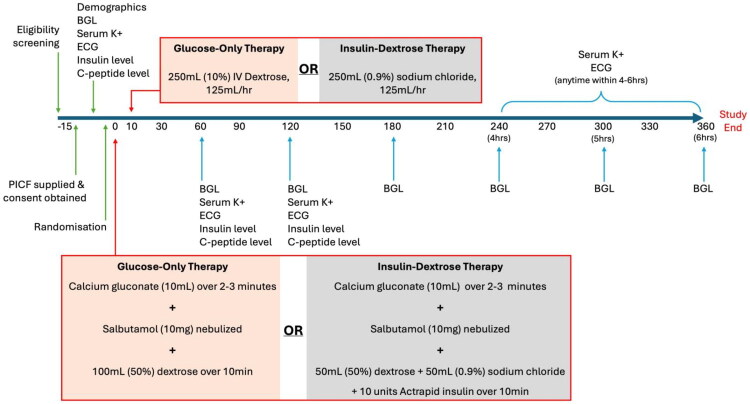
HIGH K study timeline; BGL, blood glucose level; ECG, Electrocardiography; IV, intravenous; PICF, patient information & consent form.

All outcomes will be determined within the 6-hour monitoring period using assessments outlined in Table 2 which are taken from bedside monitoring, laboratory results, and review of medical records. This monitoring period commences when the first bag has begun to be administered. An additional follow-up ECG will be obtained at the time the patient’s serum potassium is confirmed to be <5.2 mmol/L (93 mg/dL), which may occur within or after the 6-hour study period, to help differentiate hyperkalaemia-related ECG changes from baseline findings. The overall study timeline is outlined in Figure 2 below.

**Table 2.** HIGH K study assessments.

	Screening	Baseline	Hour 1	Hour 2	Hour 3	Hour 4	Hour 5	Hour 6	Post Discharge from ED
Eligibility	X	—	—	—	—	—	—	—	—
Screening	X	—	—	—	—	—	—	—	—
Randomisation	—	X	—	—	—	—	—	—	—
Demographics & baseline data	—	X	—	—	—	—	—	—	—
Comorbidities & medicines	—	X	—	—	—	—	—	—	—
BGL	—	X	X	X	X	X	X	X	—
Serum K^+^	—	X	X	X	—	—	X*	—	—
ECG	—	X	X	X	—	—	X*	—	X
Insulin level	—	X	X	X	—	—	—	—	—
C-peptide level^†^	—	X	X	X	—	—	—	—	—
Medications administered	—	—	—	—	—	—	—	—	X
Discharge destination	—	—	—	—	—	—	—	—	X
Protocol deviations	—	—	—	—	—	—	—	—	X
Adverse events	—	—	—	—	—	—	—	—	X

* Can be taken anytime within 4—6 h. ED = emergency department, BGL = blood glucose level, ECG = electrocardiograph.

^†^ C-peptide measurement was introduced as a protocol amendment shortly after trial commencement; data are available for all subsequent enrollments.

### Outcomes

The primary safety outcome is the incidence of hypoglycaemia (blood glucose <3.9 mmol/L (70 mg/dL), within 6 h post-treatment). Blood glucose is measured using validated bedside point-of-care glucometers. The primary non-inferiority outcome is the mean change in serum potassium (mmol/L) from baseline to 2 h post-treatment. Note that if a patient is enrolled based on a blood gas result due to clinical urgency, only the contemporaneous serum sample will be used for analysis. Secondary outcomes are severity of hypoglycaemia (ADA criteria); incidence of infusion-related complications including thrombophlebitis and extravasation; proportion of participants requiring rescue insulin therapy within 6 h post-treatment; mean change in serum potassium from baseline at 1 h and 4–6 h post-treatment; proportion of participants achieving a clinically significant potassium reduction (≥0.5 mmol/L [9 mg/dL] within 2 h); presence and resolution of hyperkalaemic ECG changes; serum insulin and C-peptide concentrations at baseline, 1 h, and 2 h post-treatment.

### Trial feasibility, challenges and limitations

Recruitment and enrolment in this study are inherently complex due to the time-critical nature of hyperkalaemia management. Clinicians must rapidly identify eligible patients and navigate a narrow treatment window while ensuring patients have adequate opportunity to provide informed consent—a task made difficult by the high-acuity ED environment. These clinical challenges are further compounded by the pathophysiological characteristics of this cohort; specifically, patients with end-stage renal disease (ESRD) often present with difficult venous access. The subsequent need for reliable, large-bore intravenous access to safely administer hypertonic glucose infusions can introduce significant delays to the enrolment process.

Beyond these clinical hurdles, the emergency setting is characterized by a pressured workload and rapid patient movement. Frequent transfers to inpatient wards or dialysis units act as a barrier to complete data collection, as maintaining the protocol’s structured follow-up and timely blood sampling becomes logistically difficult across different clinical teams and locations. Furthermore, study blinding and infusion preparation rely heavily on clinical pharmacists, effectively limiting recruitment to the hours of pharmacist availability (07:00–23:00). As a single-site RCT with a target sample size that closely approximates the expected number of eligible presentations over two years, there is limited tolerance for missed enrolments, necessitating sustained vigilance.

To mitigate these pressures, a comprehensive education and engagement campaign was undertaken prior to trial commencement. Research staff presented the protocol at departmental meetings, registrar teaching, and nursing in-services to ensure familiarity with eligibility and referral processes. Crucially, this effort is underpinned by a robust departmental research culture fostered by the successful delivery of previous randomised trials. The sustained support of the senior leadership team has been instrumental in integrating research into standard clinical workflows, ensuring that trial activity is viewed as a core component of departmental practice rather than an optional addition to a busy shift.

A further challenge and inherent limitation to the trial design is the potential for unblinding of treating clinicians, as divergent total glucose loads between arms (75 g glucose-only vs 25 g IDT) produce differences in blood glucose trajectories. The risk of resultant bias is, however, mitigated by the narrow window between treatment initiation and availability of the two-hour primary potassium endpoint, limiting the opportunity for any perceived glucose trend to meaningfully influence clinical behaviour or outcome ascertainment.

Finally, we note that clinical practice regarding insulin dosing for hyperkalaemia in ESRD is evolving, with some departments using 5 units rather than 10 units of short-acting insulin to mitigate hypoglycaemia risk. The IDT arm uses 10 units, reflecting the Queensland Health state-wide guideline current at both the time of protocol design and the time of writing. Generalisability of the safety comparison to settings using reduced-dose insulin regimens in ESRD should be considered when interpreting trial results.

## Statistical considerations

The target sample size for this study is 95 enrolments (approximately 48 per treatment arm). This sample size was primarily determined to provide 80% power to detect a statistically significant difference in the incidence of hypoglycaemia, assuming an incidence of 17% in the IDT group and 0% in the glucose-only group, using a two-sided significance level (α) of 0.05.

To account for repeat enrolments (maximum of two per participant), a design effect of 1.1 was applied. This was derived from an assumed intra-class correlation coefficient (ICC) of 0.3 and an average of 1.2 enrolments per participant. The ICC was conservatively selected to reflect potential within-subject correlation in hypoglycaemia risk. The final figure of 95 enrolments includes a 10% buffer to account for attrition or missing data (e.g. missed blood glucose measurements or haemolysed potassium samples).

This sample size also provides sufficient power for the primary non-inferiority objective serum potassium reduction at two hours. With a non-inferiority margin of −0.5 mmol/L (–9 mg/dL), an assumed true mean difference of 0.0 mmol/L and a standard deviation of 0.8 mmol/L (14.4 mg/dL), a minimum of 82 enrolments is required to achieve 80% power (α = 0.025, one-sided). Thus, the planned recruitment of 95 enrolments ensures the study is adequately powered for both primary endpoints.

All analyses will adhere to a modified intention-to-treat (mITT) principle, including participants who are randomised, receive the assigned intervention, and have at least one post-baseline potassium and BGL measurement. For participants commencing dialysis within the 6-hour observation period, data up to the point of dialysis initiation will be included. Per-protocol analyses will be undertaken as sensitivity analyses to assess potential bias arising from treatment cross-over (patients in the glucose only group receiving insulin).

For the primary safety outcome (hypoglycaemia incidence), proportions will be summarised by treatment group, and the between-group difference will be reported with 95% confidence intervals (CI). Groups will be compared using Fisher’s exact test. If clustering due to repeat enrolments is evident, a generalised linear model with a binomial error structure and robust standard errors adjusted for clustering will be used.

For the primary efficacy outcome (change in serum potassium at two hours), mean (SD) reductions in each group and the between-group mean difference (95% CI) will be reported. Non-inferiority will be concluded if the lower bound of the 95% CI is greater than −0.5 mmol/L (–9 mg/dL).

Secondary outcomes will be summarised descriptively by treatment group. To compare groups, general linear models will be calculated with an identity or binomial error structure for continuous or categorical outcomes. Group differences will be reported as mean differences (continuous) and risk differences or risk ratios (categorical) with 95% CIs. Repeated measures data will be analysed using linear mixed-effects models, which account for within-subject correlation and accommodate missing data.

Missing data will be managed based on the extent of data loss. If missingness is minimal (<5%), cases with missing data will be excluded from analysis. Potassium results from haemolysed samples will be counted as missing data. Mixed-effects modelling will allow the inclusion of all available data, mitigating bias related to incomplete measurements.

No adjustment for baseline covariates is planned, as randomisation is expected to achieve balance between treatment groups. A two-sided p-value of <0.05 will be considered statistically significant. All analyses will be performed using an appropriate statistical software package.

## Data and trial monitoring

A formal Data Monitoring Committee with pre-specified interim efficacy analyses was not implemented due to the small size of the trial, making statistical stopping rules of limited utility. An independent safety committee has been appointed to review unexpected serious adverse events deemed related to study interventions or other safety signals requiring urgent review.

The research team will conduct monitoring and quality assurance activities to ensure compliance with the approved protocol, ethical standards, and institutional guidelines. These include routine accuracy checks, source verification in the REDCap database, and ongoing monitoring of recruitment and retention metrics. Study data will be stored in a password-protected REDCap database, while scanned study forms will be kept in a secure Queensland Health drive folder, with access restricted to the research team.

## Ethics and dissemination

The trial is an investigator-initiated study. The lead investigators, including those affiliated with the University of Queensland and the Royal Brisbane and Women’s Hospital, were responsible for all aspects of trial design and execution including data collection, analysis, and decisions regarding publication. Ethical approval has been obtained from the Metro North Human Research Ethics Committee (HREC ID: 115385). The study adheres to the principles stated in the Declaration of Helsinki.

## Potential impact and dissemination

The primary potential impact of this study is to determine whether glucose-only therapy can serve as an insulin-sparing treatment option for hyperkalaemia in patients without diabetes. By eliminating the need for exogenous insulin in this population, glucose-only therapy removes the risk of iatrogenic hypoglycaemia—a complication that remains a major safety concern with standard insulin–dextrose therapy, particularly in patients without diabetes. If demonstrated to be both safe and biochemically effective, this approach could potentially improve patient safety and reshape current ED practice.

Beyond individual patient outcomes, the adoption of glucose-only therapy could deliver broader operational advantages. It does not require refrigeration or intensive post-administration glucose monitoring, enhancing its practicality in busy ED and improving workflow efficiency. These features also make it well suited to pre-hospital or resource-limited environments, where access to insulin, refrigeration, or frequent glucose monitoring may be constrained. These principles may have relevance in battlefield or remote settings, where prolonged evacuation and logistical constraints compound the risk of hyperkalaemia in casualties with crush injury or ischaemia-reperfusion injury following tourniquet release [23]. However, the substantial counter-regulatory hormone response to haemorrhagic shock may significantly impair endogenous insulin secretion, undermining the core mechanism and so further research in this population is therefore required before extrapolation can be justified [21]. Regardless of results, findings will be disseminated in peer-reviewed journals and at national and international conferences.

## Supplementary Material

SPIRIT 2025 HIGH K v2.docx

## Data Availability

Datasets will be made available upon reasonable request to the corresponding author.

## Patient and public involvement

Patients and the public were not involved in the design, conduct, reporting, or dissemination plans of this research protocol.

## Trial status

Participant enrolment began in November 2025. As of the time of publication, 24 patients have been enrolled in the study representing 25% of the intended sample size. The last patient is expected to be recruited in September 2027, with final study completion anticipated in November 2027.
